# Identification of a novel CNV at the *EYA4* gene in a Chinese family with autosomal dominant nonsyndromic hearing loss

**DOI:** 10.1186/s12920-022-01269-x

**Published:** 2022-05-16

**Authors:** Weixun Zhang, Jing Song, Busheng Tong, Mengye Ma, Luo Guo, Yasheng Yuan, Juanmei Yang

**Affiliations:** 1 Department of Otology and Skull Base Surgery, Eye Ear Nose and Throat Hospital, Fudan University, Shanghai, 200031 China; 2 Shanghai Clinical Medical Center of Hearing Medicine, Shanghai, 200031 China; 3 Key Laboratory of Hearing Medicine of National Health Commission of the People’s Republic of China, Shanghai, 20031 China; 4 Research Institute of Otolaryngology, Fudan University, Shanghai, 200031 China; 5 Lateral Skull Base Diagnosis and Treatment Center, Eye Ear Nose and Throat Hospital, Fudan University, Shanghai, 200031 China; 6 Department of Otorhinolaryngology Head and Neck Surgery, First Affiliated Hospital of Anhui Medical University, Jixi Road 218, Hefei, 230022 Anhui China; 7 ENT Institute and Department of Otorhinolaryngology, Eye & ENT Hospital, Fudan University, 83 Fenyang Road, Xuhui District, Shanghai, China

**Keywords:** *EYA4*, DFNA10, Copy number variation (CNV), Deafness, Whole genome sequencing (WGS)

## Abstract

**Background:**

Hereditary hearing loss is a heterogeneous class of disorders that exhibits various patterns of inheritance and involves many genes. Variants in the *EYA4* gene in DFNA10 are known to lead to postlingual, progressive, autosomal dominant nonsyndromic hereditary hearing loss.

**Patients and methods:**

We collected a four-generation Chinese family with autosomal-dominant nonsyndromic hearing loss (ADNSHL). We applied targeted next-generation sequencing (TNGS) in three patients of this pedigree and whole-genome sequencing (WGS) in the proband. The intrafamilial cosegregation of the variant and the deafness phenotype were confirmed by PCR, gap-PCR and Sanger sequencing.

**Results:**

A novel CNV deletion at 6q23 in exons 8–11 of the *EYA4* gene with a 10 bp insertion was identified by TNGS and WGS and segregated with the ADNSHL phenotypes**.**

**Conclusions:**

Our results expanded the variant spectrum and genotype‒phenotype correlation of the *EYA4* gene and autosomal dominant nonsyndromic hereditary hearing loss in Chinese Han individuals. WGS is an accurate and effective method for verifying the genomic features of CNVs.

**Supplementary Information:**

The online version contains supplementary material available at 10.1186/s12920-022-01269-x.

## Introduction

Hearing loss is the most common sensory deficit in modern society and affects approximately 466 million people worldwide, 34 million of whom are children [1, 2]. Hereditary hearing loss patterns vary among autosomal dominant, autosomal recessive, X-linked, and mitochondrial patterns of inheritance. Unlike syndromic hearing losses, non-syndromic hearing loss are not associated with other clinical abnormalities [3]. In 75–80% of cases, NSHL is inherited autosomally recessively, while 20% are inherited autosomally dominantly. X-linked (2–5%) or mitochondrial patterns of inheritance (1%) are rare in NSHL [3, 4]. The main characteristics of autosomal dominant hearing loss are high genetic and clinical heterogeneity and a delayed onset (postlingual), which are easily overlooked during hearing screening of newborns. At present, 51 different genes and 67 different loci have been linked to autosomal-dominant NSHL [5].

The *EYA4* gene, which is located on chromosome 6q22.3-q23.2, was first identified as a causal gene of DFNA10 in a large American family in 1996 [6]. The *EYA4* gene encodes eye absent 4 protein and is considered necessary for the proper development of multiple human organs, including the eye, inner ear, and heart. The EYA4 protein comprises two functional domains that contain a 271-amino-acid highly conserved C-terminal EYA domain (eyaHR) and an N-terminal variable region with a proline-serine-threonine (PST)-rich transactivation domain (eyaVR) [7]. By mediating interactions with the sine oculis family of proteins (Six1–Six6), mammalian EYA proteins function as transcriptional coactivators [8].

Researchers from different countries have found novel variants of the *EYA4* gene and deletions of the *EYA4* allele in different families with hearing loss based on sequencing analysis [9–14]. Late-onset, progressive, sensorineural hearing loss and age of onset from 6 to 50 years are the common characteristics among the tested families. Mid-frequency hearing is affected first, and all frequencies gradually become affected with increasing age. The degree of hearing loss also ranges from mild to moderate or severe with spontaneous evolution [15].

In recent years, next-generation sequencing (NGS) technology, including both targeted and whole-genome sequencing (WGS), has been considered an efficient and swift method to detect potential variants [16, 17]. This method provides a guiding role in the diagnosis and treatment of hereditary hearing loss [18]. Copy number variations (CNVs) are genomic variants within species that reflect differences in copy numbers, including deletions, duplications, and amplifications of DNA sequences. By using cytogenomics techniques such as comparative genomic hybridization (CGH), SNP arrays, WES and WGS, many novel CNVs associated with NSHL phenotypes have been identified [19–23]. With the development of diagnostic WGS, the accessibility, robustness and accuracy of CNVs throughout the genome have dramatically improved. Compared to genomic microarrays, the investigation of genomic features such as copy number, content and positional information has become more precise through the WGS method and algorithm [24, 25].

In the present study, we present a four-generation Chinese pedigree with autosomal dominant nonsyndromic hearing loss. A novel CNV deletion at 6q23 was identified in the affected individuals by targeted next-generation sequencing (TNGS) and WGS, and this information sheds new light on the pathogenic mechanism of *EYA4* variants.

## Patients, materials and methods

### Family and clinical evaluation.

A Chinese family (FY-140) classified as of Han origin presented with late-onset, progressive hearing loss. Approval for the study was obtained from the Ethics Committee of the Eye & ENT Hospital, Fudan University for Human Studies. Written informed consent was obtained from the participants or the parents of minors. The assessment of all the individuals was based on audiological methods, including pure-tone audiometry, acoustic impedance, auditory brainstem response (ABR), distortion product of otoacoustic emission (DPOAE) and otological examination. Clinical information, such as age of onset, degree of hearing loss, progression of hearing loss, noise exposure and history of using aminoglycosides, was collected from family members if available. Information on deceased family members was obtained from relatives. The proband (II-2) was subjected to a high-resolution CT scan of the temporal bone. All the individuals accepted electrocardiography for the reason that some references indicated that EYA4 gene variants may cause heart disease in patients.

### Targeted genomic capturing and next-generation sequencing

Genomic DNA was isolated using the TIANamp Blood DNA Midi Kit (TIANGEN Biotech, Beijing China) and fragmented to 150 bp using an ultrasonoscope (Covaris S220, Massachusetts, USA). End repair, adenylation and adapter ligation were performed for library preparation using a standard library construction kit (MyGenostics Inc., Beijing, China). Targeted DNA fragments were captured by a sequence capture array (MyGenostics Inc., Beijing, China). High-throughput sequencing and processing and bioinformatic data analysis were performed using the DNBSEQ-T7 sequencing platform (MGI Tech Co, Shenzhen China). The raw sequence reads were filtered using the BWA MultiVision software package and then aligned to GRCh38/hg38 (University of California Santa Cruz version). SNPs and indels were identified using the GATK Indel Genotyper and ANNOVA software. A CNV analysis was performed using the log2 ratio of the read depth on each exon.

### Whole-genome sequencing

The genomic DNA samples were fragmented by sonication to a size of 300–500 bp. Sequence analysis was performed using the TruSeq Nano DNAHT Sample Prep Kit (Illumina Inc., Massachusetts, USA) following the manufacturer’s instructions. The total effective data yield of the sample was approximately 430 million reads, and the data showed a coverage of > 99.62% at 20X. After the raw sequence reads were mapped to the human genome reference sequence (USSC) (GRCh38/hg38), SpeedSeq software was used. SNPs, indels, CNVs and SVs were captured using GATK HaplotypeCaller, ANNOVAR and SpeedSeq software.

### PCR, gap-PCR and Sanger sequencing

PCR, gap-PCR and Sanger sequencing were performed to analyze the cosegregation of variants with NSHL in this family. All primers were designed with Primer3 software (Applied Biosystems). Gap-PCR was designed to detect certain CNVs of the *EYA4* gene. The following seven PCR primers were used for analysis of the suspected variants: *EYA4* gene (Forward1, 5′-ATGAAGCCAAACACATATATTTCAA-3′; Forward2, 5′-TAGTGGCTACAGCCCCAGATCA-3′; and Reverse, 5′-AAACATTTTGGATGACGTTCCAT-3′) and *CDH23* gene (Forward1, 5′-CACCCAGGTGGTGATCCAAGT-3′; Reverse1, 5′-GGAGCAGGAGAGTAGCTCTGGTTG-3′; Forward2, 5′'-CAGTACCAGCTGCTGACAGTGC-3′; and Reverse2, AGCAGGGCATATGTGGGTCATCT-3′). The cycling program was as follows: 95 °C for 2 min; 11 cycles of 94 °C for 20 s, 64–0.5 °C per cycle for 40 s, and 72 °C for 1 min; 24 cycles of 94 °C for 20 s, 58 °C for 30 s, and 72 °C for 1 min; 72 °C for 2 min; and 4 °C for the rest of the time. The standard protocols for Sanger sequencing were performed using an ABI 3730XL Dx Genetic AnalyserAnalyzer (Applied Biosystems) and PolyPhred software to confirm the detected variants in patients (II-2, II-3, II-6 and III-1) and healthy family members (I-2, III-2, III-3, III-5, IV-1 and IV-2).

## Results

### Clinical evaluation

**Fig. 1:**
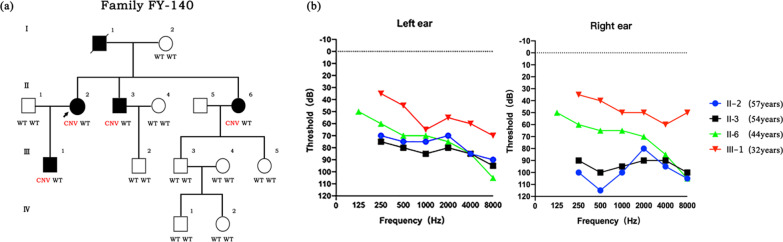
**a** Pedigree diagram of the four generations of FY-140 with ADNSHL. **b** Audiogram curves of the 4 affected members of FY140. Right ear tonal audiometric curves showing that the audiogram of III-1 exhibits a ‘cookie-bite’ pattern

The pedigree of the family includes 15 members (eight men and seven women) over four generations (Fig. 1A). Five individuals were diagnosed with NSHL based on their medical history and audiological function examination results. The self-reported age of onset of hearing impairment ranged from 26 to 43 years. Assessments of the four affected living members showed mild to severe bilaterally symmetric NSHL across all frequencies, and the disease affected both sexes (Table 1). In addition to hearing loss, the patients had no other clinical symptoms or signs. No patients complained of vestibular symptoms. The temporal bone scans and cardiac examinations of the proband yielded normal results. The initial hearing loss showed an audiogram pattern called a “cookie bite”, which was usually mild and only affected mid-frequencies. Progressive hearing loss expanded to other frequencies at later stages (Fig. 1B). Other audiometric tests' results in affected individuals including ABR, DPOAE, acoustic impedance and otological examination were also consistent with the diagnosis of NSHL.

**Table 1:** Summary of the phenotypic information of the family members

Subjects	Gender	Age (years)		Pure-tone average (dBHL)		Audiogram shape	Degree of hearing loss
		At testing	At onset	Left	Right		
I-1	Male	76	–	82	90	–	Severe
I-2	Female	72	–	Normal	Normal	–	Normal hearing
II-1	Male	62	–	Normal	Normal	–	Normal hearing
II-2	Female	57	42	77	98	Flat	Moderate–severe
II-3	Male	54	43	83	94	Flat	Severe
II-4	Female	51	–	Normal	Normal	–	Normal hearing
II-5	Male	53	–	Normal	Normal	–	Normal hearing
II-6	Female	44	37	75	71.25	Flat–sloping	Moderate–severe
III-1	Male	32	26	55	45	Cookie–bite	Moderate
III-2	Male	30	–	Normal	Normal	–	Normal hearing
III-3	Male	27	–	Normal	Normal	–	Normal hearing
III-4	Female	26	–	Normal	Normal	–	Normal hearing
III-5	Female	26	–	Normal	Normal	–	Normal hearing
IV-1	Male	4	–	Normal	Normal	–	Normal hearing
IV-2	Female	2	–	Normal	Normal	–	Normal hearing

### Identification of novel CNV by TNGS

**Fig. 2:**
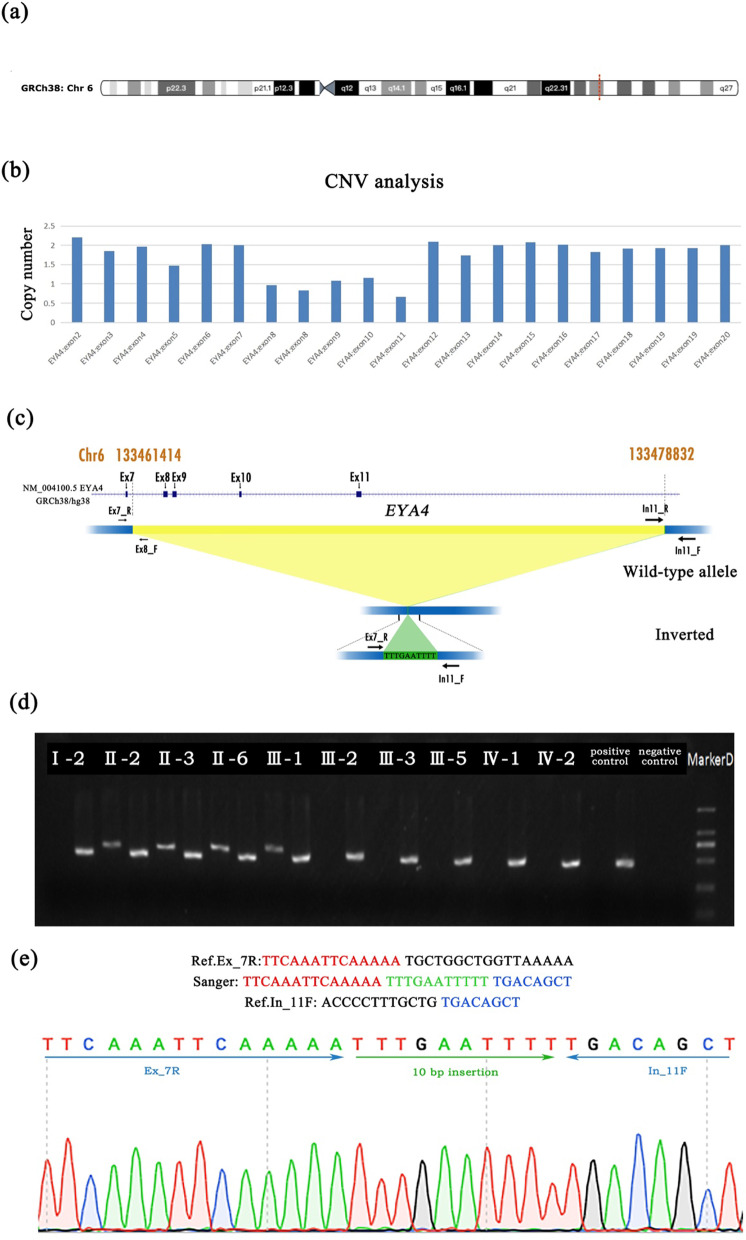
Identification of a novel copy number variation in the *EYA4* gene in a Chinese family. **a** Schematic diagram showing the position of the *EYA4* gene deletion on chromosome 6. The red bar indicates the alignment position of the deletion. **b** Copy number of each exon calculated from the fluorescence peak ratios identified from the CNV analysis. **c** Scheme of the normal and inverted alleles. **d** Gap-PCR product of the distal breakpoint junction showing segregation with the phenotype in the family. **e** Sanger sequencing of the inverted allele by *EYA4*_Ex7_F and *EYA4*_In11_R covering the two breakpoints and a 10-bp insertion

Patients II-2, III-1, and III-5 were subjected to targeted NGS of 147 deafness-related genes, and 8 SNPs and 1 CNV were detected. A novel CNV in exons 8–11 of the *EYA4* gene and two previously identified variants (c.7630T > G, p. Leu2544Val and c.8257G > A p. Ala2753Thr) in the *CDH23* gene were found in all three patients (Fig. 2A, B). The intrafamilial cosegregation of the variants and the hearing loss phenotype were confirmed by long-range PCR and Sanger sequencing in all family members.

We found that both variants of the *CDH23* gene (NM_022124), c.7630T > G (p. Leu2544Val) in exon 52 and c.8257G > A (p. Ala2753Thr) in exon 54, were been carried by four patients (II-2, II-3, II-6 and III-1) and five healthy family members (I-2, III-2, III-3, III-5, IV-1 and IV-2) by Sanger sequencing. These four patients and five healthy family members are all heterozygous for both variants of the *CDH23* gene. According to the pedigree diagram, the inheritance pattern of the hearing loss in this family was dominant. Both variants of the *CDH23* gene did not co-segregate with the phenotype (BS4-ACMG).The genotype for each variant in the pedigree and the Sanger sequencing results of these family members are described in the supplemental material (Additional file 1: Fig. S1, Additional file 2: Fig. S2, Additional file 3: Fig. S3). We believe they are in cis-mutation and not in compound heterozygosity in each subject. Although they have been reported in a previous paper that detected both variants in a deafness patient from China, these variants did not appear in the general populations databases [26]. The minor allele frequency (MAF) of p. Leu2544Val variant is 0.000051 (East Asian) in GnomAD, while the p. Ala2753Thr variant is novel (PM2-ACMG). These variants were predicted as benign by silico pathogenicity prediction tools REVEL, MutationTaster, SIFT and Polyphen 2 (BP4-ACMG). According to ACMG standards and guidelines, both variants in the *CDH23* gene are classified as PM2, BP4 and BS4, and the criteria for both alleles will be of uncertain significance (Table 2). In general, *CDH23* gene mutations cause autosomal recessive non-syndromic hearing loss [4, 27]. Based on the ACMG classification and intrafamilial cosegregation analysis of these variants, we inferred that the c.7630T > G and c.8257G > A variants in the *CDH23* gene may not be the cause of the disease in this family. In contrast, a CNV in the *EYA4* gene was found only in the patients, and none of the healthy family members were carriers of this deletion (Fig. 2C). Therefore, this variant in the *EYA4* gene could be considered the cause of the disease. Because patient I-1 died, we were unable to collect a sample of his DNA. We inferred that patients II-2, II-3 and II-6 inherited the heterozygotic variant from their father and that patient III-1 inherited the variant from patient II-2.

**Table 2:** Identified *CDH23* variant and in silico molecular genetic analysis

Gene name	Transcript accession number	cDNA change	Protein change	Position (GRCh38/hg18)	REVEL	MutationTaster	SIFT	Polyphen 2	ACMG Classification
*CDH23*	NM_0221 24;exon52	c.7630T > G	p.Leu2544Val	chr10:71803045	Benign (0.025)	Disease causing (0.999999989)	Benign (0.393)	Benign (0.002)	Uncertain Significance (PM2 + BP4 + BS4)
*CDH23*	NM_0221 24;exon56	c.8257G > A	p.Ala2753Thr	chr10:71807355	Benign (0.363)	Disease causing (0.999999778)	Benign (0.01)	Benign (0.032)	Uncertain Significance (PM2 + BP4 + BS4)

### Verification of CNV by WGS

We performed WGS using genomic DNA from patient III-1 to further identify potential variants. We identified on average 3,585,580 SNPs and 732,397 indels in coding regions or introns. Then, Single-nucleotide variants (SNVs) and InDel from WGS data were filtered as follows: (1) variants with MAF below 0.01 in 1000Genomes, ExAC03 Asian population and gnomAD Asian population (2) coding/splicing variants (3) variants that are predicted to be likely pathogenic/pathogenic by any of the following software such as SIFT, POLYPhen V2, MutationTaster, Cadd, Dann, and dbscSNV, were considered as likely causal variants. CNV analysis was performed using the log2-ratio of read depth on each exon. Priority was given to variants found in deafness genes (annotated as deafness genes in one or more of the following databases: OMIM, HPO, HGMD, InterVar, HPO, MGI, ClinVar, ISCA and MalaCards). There were 64 variants predicted as candidates, including 47 SNVs, 3 indels, and 2 CNVs. With WGS, TNGS, Sanger sequencing, and cosegregation analysis, we identified a novel CNV that might cause disease.

The complex genomic rearrangement in the dataset encompassed a 17.4-kb deletion spanning exons 8 to 11 and a 10-base insertion (chr6: 133461414 insTTTGAATTTT). As shown in Fig. 2, the distal breakpoint mapped to chr6: 133461414 (GRCh38/hg38), and the proximal breakpoint mapped to chr6: 133478832 (GRCh38/hg38) (Fig. 2C). To validate the identified CNV, gap-PCR of the region spanning the breakpoint was performed. The primers Forward1 and Reverse amplify a gap-PCR product specific for the variant allele, whereas the primers Forward2 and Reverse amplify a product from the normal *EYA4* gene. The 500-bp PCR product was obtained from all 4 affected individuals (II-2, II-3, II-6 and III-1) in the family but not from the unaffected family members (I-2, III-2, III-3, III-5, IV-1 and IV-2). The normal *EYA4* gene product was found in all family members (Fig. 2D). The generated PCR products were subjected to Sanger sequencing, and a sequence analysis of the resultant amplicons confirmed the junction of the two breakpoints through the 10-bp insertion identified by WGS (Fig. 2E). This deletion has been submitted to LOVD under accession ID 00361730.

## Discussion

CNVs are a common cause of hereditary hearing loss and are thought to play a role in nearly 20% of non-syndromic HL diagnoses [19]. In this study, we performed a comprehensive genetic analysis that included TNGS, WGS, gap-PCR and Sanger sequencing in a four-generation Chinese Han family with autosomal dominant NSHL. All the affected individuals in the family exhibited sensorineural deafness, which primarily affected low and mid-frequencies and had onset ages in the range of 26 to 43 years.

We identified a novel CNV deletion in exons 8–11 of the *EYA4* gene with a 10 bp insertion. First, this variant cosegregated with NSHL symptoms in patients and was not detected in normal family members. Then, this CNV is predicted to affect the eyaHR domain. By interacting with members of the SIX and DACH protein families in a conserved network, the highly conserved C-terminal region of EYA4 (eyaHR) regulates embryonic development and follow-up functions after development of the mature organ of Corti. It regulates Na+/K+-ATPases and the development of mechanosensory cells of the inner ear [12]. Finally, we attempted to construct a three-dimensional structure of the CNV using the SWISS-MODEL software but failed because the structure was severely affected. These findings may also suggest that this novel CNV deletion is pathogenic in auditory function.

The hearing loss phenotype in the present family is similar to that reported for patients carrying *EYA4* variants, i.e., late-onset, postlingual, progressive, and bilateral HL. In previous reports, flat-type hearing loss was observed in patients with truncating *EYA4* variants. At onset, hearing impairment was usually mild and detected at mid-frequencies, resulting in an audiometric profile commonly referred to as a “cookie-bite” pattern. During its progression, hearing loss began to involve other frequencies. The progression rate of hearing loss caused by *EYA4* was approximately 5.75 dB/year (95% CI 4.50–7.00 dB/year), which is relatively severe compared to *POU4F3* and *MYO6* gene mutation in ADNSHL patients [28, 29].

To date, variants in the *EYA4* gene have been associated with HL in more than 50 ethnic groups worldwide. It was believed that *EYA4* variants led to syndromic and non-syndromic NSHL, but *EYA4* is not a frequently mutated gene in ADNSHL compared with other reported genes. The characteristics of all known *EYA4* variants are summarized in Table 3. According to these variants, the severity of hearing loss was not significantly related to the types or locations of variants.

**Table 3:** Summary of all known *EYA4* variants and their hearing loss phenotypes

Variant type	Nucleotide Change	Exon/Intron	Amino Acid Change	Origin	Age at HL on set	HL degree	Audiogram profile	References
Splicing	c.84-2A > G	Intron 3		Chinese Indian	N/A	N/A	N/A	Chen et al. 2016 [36] Panigrahi et al. [37]
Missense	c.152C > T	Exon 4	p.Ser51Phe	America	N/A	N/A	N/A	Sloan-Heggen et al. [38]
Nonsense	c.160G > T	Exon 4	p.Glu54*	Spanish	42 years	Mild	MF/flat	Morín et al. [13]
Frameshift	c.222_223del	Exon 5	p.Val75Phefs*32	Japanese	61 years	Mild to moderate	HF/LF	Shinagawa et al. [9]
Frameshift	c.464delC	Exon 8	p.Pro155Glnfs*43	Swedish Dutch	N.A Childhood	N.A Moderate	N.A MF/HF	Neveling et al. [39] Van Beelen et al. [40]
Frameshift	c.498del	Exon 8	p.Thr167Leufs*31	Japanese	13 years	Mild	LF	Shinagawa et al. [9]
Missense	c.511G > C	Exon 8	p.Gly171Arg	Chinese	6–50 years	Mild to severe	HF/flat	Liu [15]
Nonsense	c.517C > T	Exon 8	p.Gln173*	Japanese	48 years	Moderate	Flat	Shinagawa et al. [9]
Missense	c.543C > G	Exon 8	p.Tyr181Ter	Chinese	Second to the fourth decade	Severe to profound	Flat	Mi et al. [32]
Frameshift	c.579_580insTACC	Exon 8	p.Asp194Tyrfs*52	Swedish	4–40 years	Mild to profound	N/A	Frykholm et al. [41]
	c.580 + 1G > A	Intron 8		Japanese	45 years	Moderate	Flat	Shinagawa et al. [9]
Frameshift	c.614dupA	Exon 9	p.Glu205Argfs*40	Chinese	20–40 years	Moderate to profound	HF/flat	Huang et al. [42]
Frameshift	c.781del	Exon 10	p.Thr261Argfs*34	Spanish	26–44 years	Mild to moderate	Gently downsloping	Morín et al. [13]
Missense	c.804G > C	Exon 10	p.Gln268His	Slovak	10–40 years	Moderate	Gently downsloping	Varga et al. [43]
Nonsense	c.863C > A	Exon 11	p.Ser288*	Korean Korean	N.A N.A	Moderate Moderate to severe	Reverse U-shaped Flat	Baek et al. [44] Kim et al. [10]
Missense	c.866C > T	Exon 11	p.Thr289Met	American	N.A	N.A	N.A	Miszalski-Jamka et al. [45]
Frameshift	c.910del	Exon 11	p.Ser305Leufs*15	Japanese	30 years	Severe	Flat	Shinagawa et al. [9]
Missense	c. 978C > G	Exon 12	p.Phe326Leu	Korean	N.A	Moderate	Down sloping	Choi et al. [46]
Nonsense	c.988C > T	Exon 12	p.Gln330*	Japanese	16 years	Moderate	Flat	Shinagawa et al. [9]
Frameshift	c.1026_1027dupAA	Exon 12	p.Thr343Lysfs*62	American	N.A	Moderate to profound	Flat/Gently sloping	Wayne et al. [12]
Frameshift	c.1048_1049dupAA	Exon 12	p.Arg352Profs*53	American	N.A	Moderate to severe	MF/HF	Makishima et al. [47]
Missense	c.1078C > A	Exon 12	p.Pro360Thr	Spanish	44 years	Mild to moderate	Gently downsloping	Morín et al. [13]
Missense	c.1107G > T	Exon 12	p.Glu369Asp	Spanish	10–11 years	Moderate to severe	Gently downsloping	Morín et al. [13]
Missense	c.1109G > A	Exon 13	p.Arg370His	Philippines	N.A	N.A	N.A	Truong et al. 2019 [48]
Missense	c.1109G > C	Exon 13	p.Arg370Pro	Japanese	30 years	Mild to moderate	MF	Shinagawa et al. [9]
Missense	c.1109G > C	Exon 13	p.Val371Met	Belgium	N.A	N.A	N.A	Sommen et al. [49]
Frameshift	c.1115_1118dup TTGT	Exon 13	p.Trp374Cysfs*6	Hungarian	N.A	N.A	N.A	Pfister et al. [50]
Missense	c.1122G > T	Exon 13	p.Trp374Cys	Australian	10–25 years	Mild to severe	Gently downsloping	Morín et al. [13]
Missense	c.1154C > T	Exon 13	p.Ser385Leu	Italian	N.A	Mild to profound	MF	Cesca et al. [51]
Nonsense	c.1177C > T	Exon 13	p.Gln393*	Korean Japanese	N.A 26 years	moderate	HF Flat	Kim [10] Shinagawa et al. [9]
Frameshift	c.1194del	Exon 14	p.Met401Trpfs*3	Korean	N.A	Moderate	Down sloping	Choi et al. [46]
Missense	c.1216G > C	Exon 14	p.Gly406Arg	Japanese	5 years	Moderate	Flat	Shinagawa et al. [9]
Missense	c.1223G > A	Exon 14	p.Arg408His	America	N.A	N.A	N.A	Miszalski-Jamka et al. [45]
Missense	c.1281G > A	Exon 14	p.Glu427Glu	Spanish	26 years	Moderate to profound	Flat	Morín et al. [13]
Splicing	c.1282-12T > A	Intron 14		Australian	N.A	Mild to profound	Flat	Hildebrand et al. [52]
Splicing	c.1282-1G > A	Intron 14		Spanish	12 years	Mild to moderate	MF/Flat	Morín et al. [13]
Missense	c.1301T > A	Exon 15	p.Ile434Lys	Chinese	8–38 years	Mild to severe	MF/flat	Tan et al. [53]
Splicing	c.1341-19T > A	Intron 15		Germany	N.A	N.A	N.A	Vona et al. [54]
Nonsense	c.1601C > G	Exon 17	p.Ser534*	Spanish	3–16 years	Moderate to severe	MF/Flat	Morín et al. [13]
Missense	c.1643C > G	Exon 18	p.Thr548Arg	Chinese	17–40 years	Mild to profound	N.A	Sun et al. [11]
Missense	c.1663G > C	Exon 18	p.Ala555Pro	Japanese	25 years	Moderate	N.A	Shinagawa et al. [9]
Splicing	c.1739-1G > A	Intron 18		America	50 years	N.A	N.A	Cirino et al. [55]
Nonsense	c.1759C > T	Exon 19	p.Arg587*	Belgian	6–40 years	Mild to moderate	N.A	Wayne et al. [12]
Frameshift	c.1790del	Exon 19	p.Val597Glyfs*4	Japanese	35 years	Moderate	Flat	Iwasa et al. [56]
Missense	c.1810G > T	Exon 19	p.Gly604Cys	Swedish Dutch	N.A	N.A	N.A	Neveling et al. [39] Van Beelen et al. [40]
Nonsense	c.1834A > T	Exon 19	p.Lys612*	Chinese	27 years	Moderate	Gently downsloping	Hu et al. [16]
Missense	c.1855T > G	Exon 20	p.Trp619Gly	Chinese	N.A	N.A	N.A	Xiao et al. [57]
CNV	Deletion 7689 bp (Ex7 to Ex11)			Japanese	25 years	Moderate to severe	LF/HF	Shinagawa et al. [9]
CNV	Deletion 9.5 Mb (Ex4 to Ex 20)			Japanese	13 years	Severe	LF/HF	Shinagawa et al. [9]
CNV	Deletion 2747 bp (Ex15 to Ex17)			Spanish	8 years	Moderate	Flat	Morín et al. [13]
CNV	Deletion 9 Mb at 6q23.1–24.1 (Ex4–20)		p.Asp194Glyfs*30	Polish	N.A	N.A	N.A	Dutrannoy et al. [30]
CNV	Deletion 4846 pb incl. intron 9, exon 10 and partial intron 10 c.581_804del (In9, Ex10, part of In10)				N.A	N.A	N.A	Schönberger et al. [34]
CNV	Deletion 10.4 Mb promoter and exon 1,2 (Ex1–2)			Japanese	20-month-old	Moderate to severe	MF/flat	Abe et al. [31]
CNV	Deletion 3.7 MB in 6q23.1q23.2 (Ex1–20)			Italian	12 years	N.A	N.A	Gana et al. [58]
CNV	Deletion 12,835 bp (Ex6–10)			Japanese	23 years	Mild to sereve	LF/HF/Flat	Ishino et al. [14]
CNV	Deletion 17.4 kb and 10 bp insertion (Ex8–11)			Chinese	26–42 years	Moderate–severe	Flat	This work

**Fig. 3:**
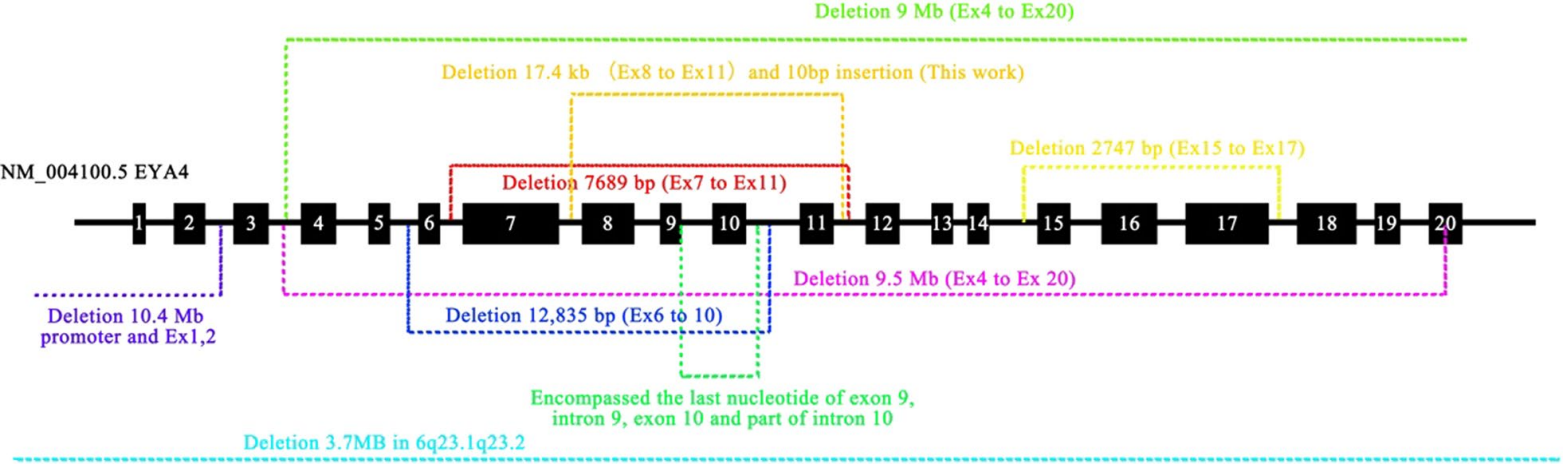
Overview of the CNVs identified in this study and those previously identified in *EYA4*

Several CNVs in *EYA4* have been linked to deafness (Fig. 3). One CNV disrupts the *EYA4* gene and spares only exons 1–3 from the deletion [30]. The deleted sequence of the promoter and the first two exons was previously identified in a Japanese boy [31]. A deletion of four exons and a deletion spanning exons 4–20 have been reported to cause severe ADNSHL in Japanese individuals [9]. In addition, the *EYA4* variant reportedly causes dilated cardiomyopathy accompanying NSHL in a single large family. In this family, a 4846-bp genomic deletion that resulted in loss of the EYA domain (eyaHR) and part of the variable region (eyaVR) was detected [32]. A heterozygous deletion of 2747 bp represented a copy variant loss encompassing exon 15 to exon 17 [13]. Additionally, in Japan, a novel hemizygous indel in the *EYA4* gene was predicted to be p. (Val124_Pro323del) [14]. We now add a genomic rearrangement consisting of a deletion and a 10-bp insertion to this list (Fig. 3, Table 3).

The *EYA4* gene is widely distributed in the inner ear, which includes otic vesicles, the Reissner membrane and the sensory epithelia of the vestibular system and Corti [12, 33]. Although the pathogenic mechanism of ADNSHL associated with *EYA4* variants remains to be further investigated, haploinsufficiency is generally thought of as the major mechanism. Many reports indicate that *EYA4* participates in important pathways in cardiac tissue. The physiological level of *Eya4* phosphatase activity is thought to participate in normal cardiac gene regulation. Instead of most DCM genes that encode structural proteins, EYA4 is a transcriptional coactivator. Large deletions comprising the variable domain are most likely to affect cardiac functions. A patient carrying a de novo 9 MB interstitial deletion that disrupts the gene EYA4 presented a patent ductus arteriosus and aortic insufficiency. A family with a 4846-bp deletion was associated with DCM as well as NSHL. In the other 2 unrelated families, polymorphic loci on chromosome 6q23 to 24 were associated with DCM and NSHL. [30, 34]. For that reason, we performed electrocardiograms for all family members. None of the members exhibited a cardiac phenotype, and electrocardiograms showed no abnormalities. Including our work, many studies indicated that the genotype–phenotype correlation of large deletions in *EYA4* and dilated cardiomyopathy was not very obvious [14]. One reason may be that previously reported cardiopathy was caused by other variants in the large deleted regions. Another reason is that the defect in a contiguous gene could account for the cardiac defects [34]. More experiments, including detecting EYA4 levels in nuclear and cytoplasmic components, may provide evidence for this theory. Increasing the yield of genetic testing among patients with both NSHL and DCM may allow for better detection of the *EYA4* gene and cardiac pathology [35].

## Conclusions

A novel CNV deletion at 6q23 in exons 8–11 of *EYA4* in a Chinese ADNSHL family was identified by WGS and Sanger sequencing. The phenotype of the family differed from that of previously reported pedigrees with CNV deletion of *EYA4* variants. The phenotypes also differed between individuals with the same variant in the same family. Our results highlight the complexity of the *EYA4* genotype and phenotype.

## Supplementary Information

**Additional file 1:** Pedigree diagram of the four generations of FY-140 with variants in CDH23.

**Additional file 2:** Sanger sequencing of c.7630T>G, p. Leu2544Val in Family FY-140.

**Additional file 3: Figure S3.** Sanger sequencing of c.8257G>A p. Ala2753Thr in Family FY-140.

## Data Availability

The authors are not able to share the clinical data due to full anonymisation of the data is very difficult. The CNV in our study was called based on the human assembly GRCh38 (https://hgdownload.soe.ucsc.edu/downloads.html#human). This CNV has been submitted to LOVD under accession ID 00361730. All sequencing data used to support the findings of this study are available from the corresponding author upon request.

## References

[1] World Health Organization (2018). Global costs of unaddressed hearing loss and cost-effectiveness of interventions.

[2] WHO: Deafness and hearing loss https://www.who.int/health-topics/hearing-loss#tab=tab_2. 20 Sept 2021.

[3] Vona B, Nanda I, Hofrichter MAH, Shehata-Dieler W, Haaf T (2015). Non-syndromic hearing loss gene identification: a brief history and glimpse into the future. Mol Cell Probes.

[4] Stelma F, Bhutta MF (2014). Non-syndromic hereditary sensorineural hearing loss: review of the genes involved. J Laryngol Otol.

[5] Van Camp G, Smith RJH: Hereditary Hearing Loss Homepage. https://hereditaryhearingloss.org. 30 Aug 2021.

[6] O'Neill ME, Marietta J, Nishimura D, Wayne S, Van Camp G, Van Laer L, Negrini C, Wilcox ER, Chen A, Fukushima K (1996). A gene for autosomal dominant late-onset progressive non-syndromic hearing loss, DFNA10, maps to chromosome 6. Hum Mol Genet.

[7] Borsani G, DeGrandi A, Ballabio A, Bulfone A, Bernard L, Banfi S, Gattuso C, Mariani M, Dixon M, Donnai D (1999). EYA4, a novel vertebrate gene related to Drosophila eyes absent. Hum Mol Genet.

[8] Ohto H, Kamada S, Tago K, Tominaga SI, Ozaki H, Sato S, Kawakami K (1999). Cooperation of six and eya in activation of their target genes through nuclear translocation of eya. Mol Cell Biol.

[9] Shinagawa J, Moteki H, Nishio S-Y, Ohyama K, Otsuki K, Iwasaki S, Masuda S, Oshikawa C, Ohta Y, Arai Y (2020). Prevalence and clinical features of hearing loss caused by EYA4 variants. Sci Rep.

[10] Kim Y-R, Kim M-A, Sagong B, Bae S-H, Lee H-J, Kim H-J, Choi JY, Lee K-Y, Kim U-K (2015). Evaluation of the contribution of the EYA4 and GRHL2 genes in Korean patients with autosomal dominant non-syndromic hearing loss. PLoS ONE.

[11] Sun Y, Zhang Z, Cheng J, Lu Y, Yang C-L, Luo Y-Y, Yang G, Yang H, Zhu L, Zhou J (2015). A novel mutation of EYA4 in a large Chinese family with autosomal dominant middle-frequency sensorineural hearing loss by targeted exome sequencing. J Hum Genet.

[12] Wayne S, Robertson NG, DeClau F, Chen N, Verhoeven K, Prasad S, Tranebjärg L, Morton CC, Ryan AF, Van Camp G (2001). Mutations in the transcriptional activator EYA4 cause late-onset deafness at the DFNA10 locus. Hum Mol Genet.

[13] Morín M, Borreguero L, Booth KT, Lachgar M, Huygen P, Villamar M, Mayo F, Barrio LC, Santos Serrão de Castro L, Morales C (2020). Insights into the pathophysiology of DFNA10 hearing loss associated with novel EYA4 variants. Sci Rep.

[14] Ishino T, Ogawa Y, Sonoyama T, Taruya T, Kono T, Hamamoto T, Ueda T, Takeno S, Moteki H, Nishio S-Y (2021). Identification of a novel copy number variation of EYA4 causing autosomal dominant non-syndromic hearing loss. Otol Neurotol.

[15] Liu F, Hu J, Xia W, Hao L, Ma J, Ma D, Ma Z (2015). Exome sequencing identifies a mutation in EYA4 as a novel cause of autosomal dominant non-syndromic hearing loss. PLoS ONE.

[16] Hu S, Sun F, Zhang J, Tang Y, Qiu J, Wang Z, Zhang L (2018). Genetic etiology study of ten chinese families with nonsyndromic hearing loss. Neural Plast.

[17] Sang S, Ling J, Liu X, Mei L, Cai X, Li T, Li W, Li M, Wen J, Liu X (2019). Proband whole-exome sequencing identified genes responsible for autosomal recessive non-syndromic hearing loss in 33 Chinese nuclear families. Front Genet.

[18] Kremer H (2019). Hereditary hearing loss; about the known and the unknown. Hear Res.

[19] Shearer AE, Kolbe DL, Azaiez H, Sloan CM, Frees KL, Weaver AE, Clark ET, Nishimura CJ, Black-Ziegelbein EA, Smith RJH (2014). Copy number variants are a common cause of non-syndromic hearing loss. Genome Med.

[20] Rosenberg C, Freitas ÉL, Uehara DT, Auricchio MTBM, Costa SS, Oiticica J, Silva AG, Krepischi AC, Mingroni-Netto RC (2016). Genomic copy number alterations in non-syndromic hearing loss. Clin Genet.

[21] Moon IS, Grant AR, Sagi V, Rehm HL, Stankovic KM (2021). TMPRSS3 gene variants with implications for auditory treatment and counseling. Front Genet.

[22] Moteki H, Azaiez H, Sloan-Heggen CM, Booth K, Nishio S-Y, Wakui K, Yamaguchi T, Kolbe DL, Iwasa Y-I, Shearer AE (2016). Detection and confirmation of deafness-causing copy number variations in the STRC gene by massively parallel sequencing and comparative genomic hybridization. Ann Otol Rhinol Laryngol.

[23] Sugiyama K, Moteki H, Kitajiri S-I, Kitano T, Nishio S-Y, Yamaguchi T, Wakui K, Abe S, Ozaki A, Motegi R (2019). Mid-frequency hearing loss is characteristic clinical feature of OTOA-associated hearing loss. Genes (Basel).

[24] Hehir-Kwa JY, Pfundt R, Veltman JA (2015). Exome sequencing and whole genome sequencing for the detection of copy number variation. Expert Rev Mol Diagn.

[25] Weischenfeldt J, Symmons O, Spitz F, Korbel JO (2013). Phenotypic impact of genomic structural variation: insights from and for human disease. Nat Rev Genet.

[26] Yang T, Wei X, Chai Y, Li L, Wu H (2013). Genetic etiology study of the non-syndromic deafness in Chinese Hans by targeted next-generation sequencing. Orphanet J Rare Dis.

[27] Miyagawa M, Nishio SY, Usami SI (2012). Prevalence and clinical features of hearing loss patients with CDH23 mutations: a large cohort study. PLoS ONE.

[28] Kitano T, Miyagawa M, Nishio S-Y, Moteki H, Oda K, Ohyama K, Miyazaki H, Hidaka H, Nakamura K-I, Murata T (2017). POU4F3 mutation screening in Japanese hearing loss patients: Massively parallel DNA sequencing-based analysis identified novel variants associated with autosomal dominant hearing loss. PLoS ONE.

[29] Miyagawa M, Nishio S-Y, Kumakawa K, Usami S-I (2015). Massively parallel DNA sequencing successfully identified seven families with deafness-associated MYO6 mutations: the mutational spectrum and clinical characteristics. Ann Otol Rhinol Laryngol.

[30] Dutrannoy V, Klopocki E, Wei R, Bommer C, Mundlos S, Graul-Neumann LM, Trimborn M (2009). De novo 9 Mb deletion of 6q23.2q24.1 disrupting the gene EYA4 in a patient with sensorineural hearing loss, cardiac malformation, and mental retardation. Eur J Med Genet.

[31] Abe Y, Oka A, Mizuguchi M, Igarashi T, Ishikawa S, Aburatani H, Yokoyama S, Asahara H, Nagao K, Yamada M (2009). EYA4, deleted in a case with middle interhemispheric variant of holoprosencephaly, interacts with SIX3 both physically and functionally. Hum Mutat.

[32] Mi Y, Liu D, Zeng B, Tian Y, Zhang H, Chen B, Zhang J, Xue H, Tang W, Zhao Y (2021). Early truncation of the N-terminal variable region of EYA4 gene causes dominant hearing loss without cardiac phenotype. Mol Genet Genomic Med.

[33] Nishio S-Y, Hattori M, Moteki H, Tsukada K, Miyagawa M, Naito T, Yoshimura H, Iwasa Y-I, Mori K, Shima Y (2015). Gene expression profiles of the cochlea and vestibular endorgans: localization and function of genes causing deafness. Ann Otol Rhinol Laryngol.

[34] Schönberger J, Levy H, Grünig E, Sangwatanaroj S, Fatkin D, MacRae C, Stäcker H, Halpin C, Eavey R, Philbin EF (2000). Dilated cardiomyopathy and sensorineural hearing loss: a heritable syndrome that maps to 6q23-24. Circulation.

[35] Ahmadmehrabi S, Li B, Park J, Devkota B, Vujkovic M, Ko Y-A, Van Wagoner D, Tang WHW, Krantz I, Ritchie M (2021). Genome-first approach to rare EYA4 variants and cardio-auditory phenotypes in adults. Hum Genet.

[36] Chen S, Dong C, Wang Q, Zhong Z, Qi Y, Ke X, Liu Y (2016). Targeted next-generation sequencing successfully detects causative genes in Chinese patients with hereditary hearing loss. Genet Test Mol Biomark.

[37] Panigrahi I, Kumari D, Anil Kumar BN (2021). Single gene variants causing deafness in Asian Indians. J Genet.

[38] Sloan-Heggen CM, Bierer AO, Shearer AE, Kolbe DL, Nishimura CJ, Frees KL, Ephraim SS, Shibata SB, Booth KT, Campbell CA (2016). Comprehensive genetic testing in the clinical evaluation of 1119 patients with hearing loss. Hum Genet.

[39] Neveling K, Feenstra I, Gilissen C, Hoefsloot LH, Kamsteeg E-J, Mensenkamp AR, Rodenburg RJT, Yntema HG, Spruijt L, Vermeer S (2013). A post-hoc comparison of the utility of sanger sequencing and exome sequencing for the diagnosis of heterogeneous diseases. Hum Mutat.

[40] van Beelen E, Oonk AMM, Leijendeckers JM, Hoefsloot EH, Pennings RJE, Feenstra I, Dieker H-J, Huygen PLM, Snik AFM, Kremer H (2016). Audiometric characteristics of a Dutch DFNA10 family with mid-frequency hearing impairment. Ear Hear.

[41] Frykholm C, Klar J, Arnesson H, Rehnman A-C, Lodahl M, Wedén U, Dahl N, Tranebjærg L, Rendtorff ND (2015). Phenotypic variability in a seven-generation Swedish family segregating autosomal dominant hearing impairment due to a novel EYA4 frameshift mutation. Gene.

[42] Huang A, Yuan Y, Liu Y, Zhu Q, Dai P (2015). A novel EYA4 mutation causing hearing loss in a Chinese DFNA family and genotype–phenotype review of EYA4 in deafness. J Transl Med.

[43] Varga L, Danis D, Skopkova M, Masindova I, Slobodova Z, Demesova L, Profant M, Gasperikova D (2019). Novel EYA4 variant in Slovak family with late onset autosomal dominant hearing loss: a case report. BMC Med Genet.

[44] Baek J-I, Oh S-K, Kim D-B, Choi S-Y, Kim U-K, Lee K-Y, Lee S-H (2012). Targeted massive parallel sequencing: the effective detection of novel causative mutations associated with hearing loss in small families. Orphanet J Rare Dis.

[45] Miszalski-Jamka K, Jefferies JL, Mazur W, Głowacki J, Hu J, Lazar M, Gibbs RA, Liczko J, Kłyś J, Venner E (2017). Novel genetic triggers and genotype–phenotype correlations in patients with left ventricular noncompaction. Circ Cardiovasc Genet.

[46] Choi BY, Park G, Gim J, Kim AR, Kim B-J, Kim H-S, Park JH, Park T, Oh S-H, Han K-H (2013). Diagnostic application of targeted resequencing for familial nonsyndromic hearing loss. PLoS ONE.

[47] Makishima T, Madeo AC, Brewer CC, Zalewski CK, Butman JA, Sachdev V, Arai AE, Holbrook BM, Rosing DR, Griffith AJ (2007). Nonsyndromic hearing loss DFNA10 and a novel mutation of EYA4: evidence for correlation of normal cardiac phenotype with truncating mutations of the Eya domain. Am J Med Genet A.

[48] Truong BT, Yarza TKL, Bootpetch Roberts T, Roberts S, Xu J, Steritz MJ, Tobias-Grasso CAM, Azamian M, Lalani SR, Mohlke KL (2019). Exome sequencing reveals novel variants and unique allelic spectrum for hearing impairment in Filipino cochlear implantees. Clin Genet.

[49] Sommen M, Schrauwen I, Vandeweyer G, Boeckx N, Corneveaux JJ, van den Ende J, Boudewyns A, De Leenheer E, Janssens S, Claes K (2016). DNA diagnostics of hereditary hearing loss: a targeted resequencing approach combined with a mutation classification system. Hum Mutat.

[50] Pfister M, Tóth T, Thiele H, Haack B, Blin N, Zenner H-P, Sziklai I, Nürnberg P, Kupka S (2002). A 4-bp insertion in the eya-homologous region (eyaHR) of EYA4 causes hearing impairment in a Hungarian family linked to DFNA10. Mol Med.

[51] Cesca F, Bettella E, Polli R, Cama E, Scimemi P, Santarelli R, Murgia A (2018). A novel mutation of the EYA4 gene associated with post-lingual hearing loss in a proband is co-segregating with a novel PAX3 mutation in two congenitally deaf family members. Int J Pediatr Otorhinolaryngol.

[52] Hildebrand MS, Coman D, Yang T, Gardner RJM, Rose E, Smith RJH, Bahlo M (2007). Dahl H-HM: a novel splice site mutation in EYA4 causes DFNA10 hearing loss. Am J Med Genet A.

[53] Tan M, Shen X, Yao J, Wei Q, Lu Y, Cao X, Xing G (2014). Identification of I411K, a novel missense EYA4 mutation causing autosomal dominant non-syndromic hearing loss. Int J Mol Med.

[54] Vona B, Müller T, Nanda I, Neuner C, Hofrichter MAH, Schröder J, Bartsch O, Läßig A, Keilmann A, Schraven S (2014). Targeted next-generation sequencing of deafness genes in hearing-impaired individuals uncovers informative mutations. Genet Med.

[55] Cirino AL, Lakdawala NK, McDonough B, Conner L, Adler D, Weinfeld M, O'Gara P, Rehm HL, Machini K, Lebo M (2017). A Comparison of whole genome sequencing to multigene panel testing in hypertrophic cardiomyopathy patients. Circ Cardiovasc Genet.

[56] Iwasa Y-I, Nishio S-Y, Usami S-I (2016). Comprehensive genetic analysis of Japanese autosomal dominant sensorineural hearing loss patients. PLoS ONE.

[57] Xiao S-Y, Qu J, Zhang Q, Ao T, Zhang J, Zhang R-H (2019). Identification of a novel missense eya4 mutation causing autosomal dominant non-syndromic hearing loss in a Chinese family. Cell Mol Biol (Noisy-le-grand).

[58] Gana S, Valetto A, Toschi B, Sardelli I, Cappelli S, Peroni D, Bertini V (2019). Familial interstitial 6q23.2 deletion including Eya4 associated with otofaciocervical syndrome. Front Genet.

