# Supplementary material for: Identification of a novel CNV at the EYA4 gene in a Chinese family with autosomal dominant nonsyndromic hearing loss
Source: BMC Med Genomics. 2022 May 16;15:113. doi: 10.1186/s12920-022-01269-x (PMC9109401; doi:10.1186/s12920-022-01269-x)
Supplement: Supplementary file 1 — Additional file 1: Pedigree diagram of the four generations of FY-140 with variants in CDH23. [file 12920_2022_1269_MOESM1_ESM.docx]

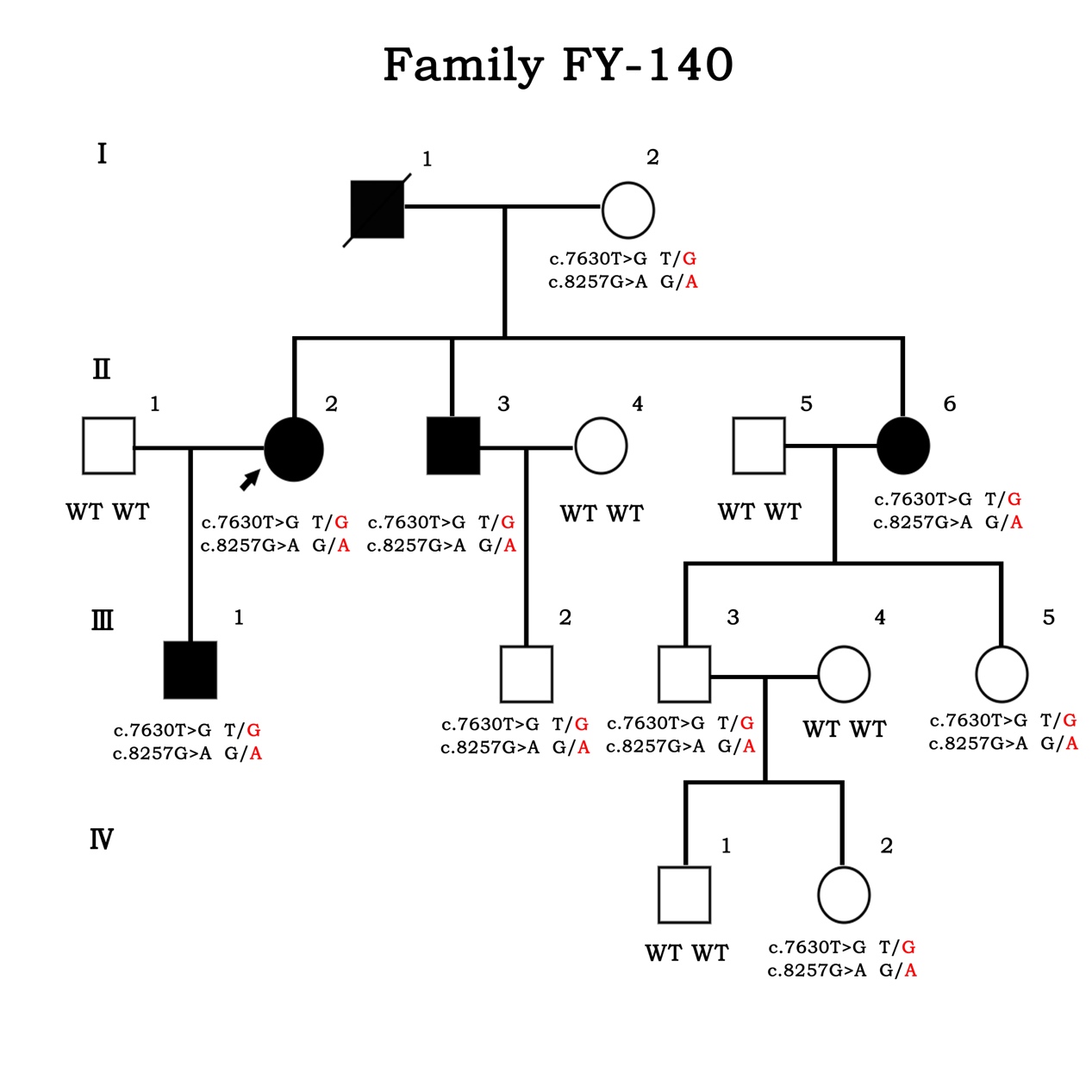


**Figure S1.** Pedigree diagram of the four generations of FY-140 with variants in *CDH23.* (c.7630T>G, p. Leu2544Val and c.8257G>A p. Ala2753Thr)
