# Supplementary figures and images for: Identification of a novel CNV at the EYA4 gene in a Chinese family with autosomal dominant nonsyndromic hearing loss
Source: BMC Med Genomics. 2022 May 16;15:113. doi: 10.1186/s12920-022-01269-x (PMC9109401; doi:10.1186/s12920-022-01269-x)

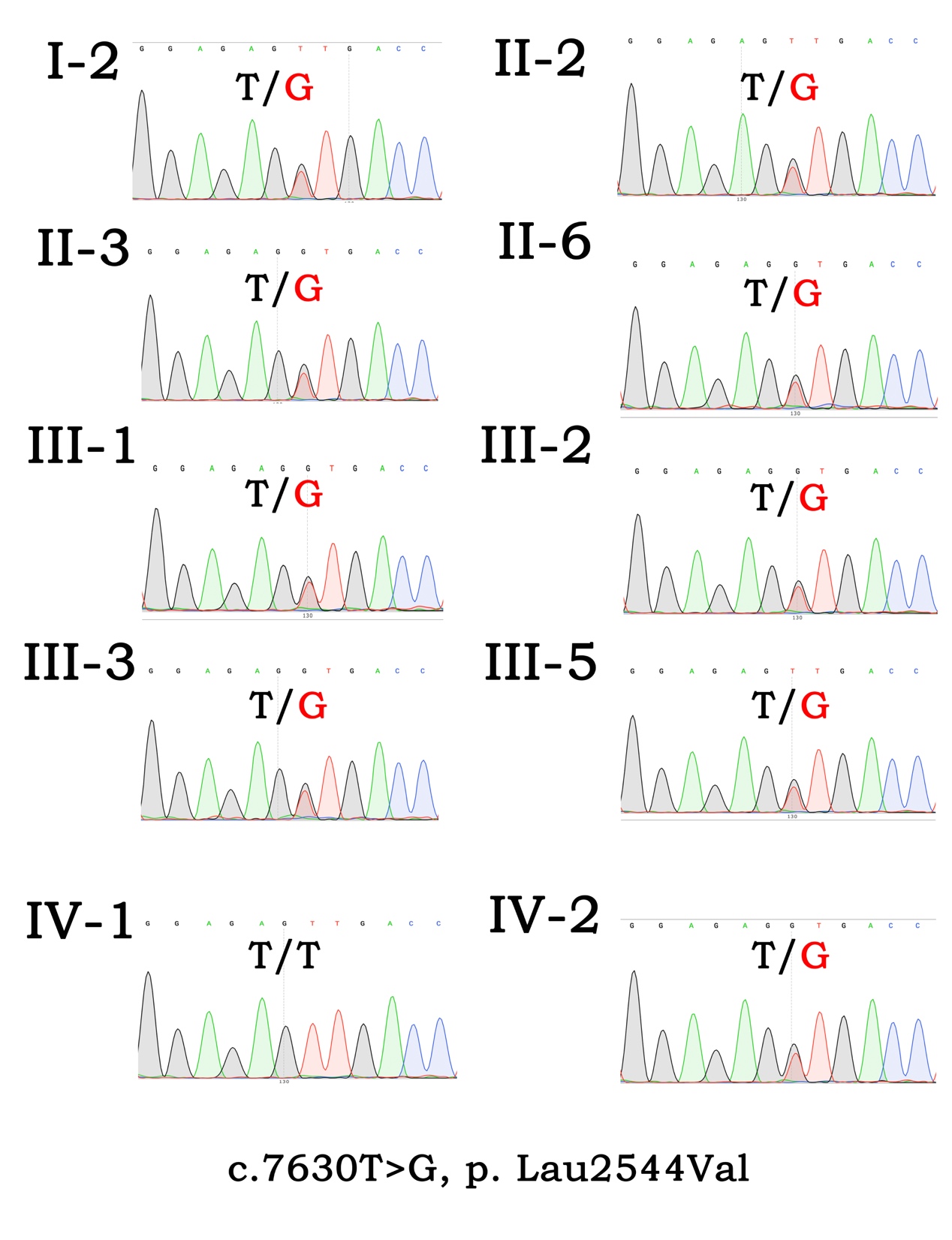


**Figure.S2** Sanger sequencing of c.7630T>G, p. Leu2544Val in Family FY‐140.

Supplement: Supplementary file 2 — Additional file 2: Sanger sequencing of c.7630T>G, p. Leu2544Val in Family FY-140. [file 12920_2022_1269_MOESM2_ESM.docx]

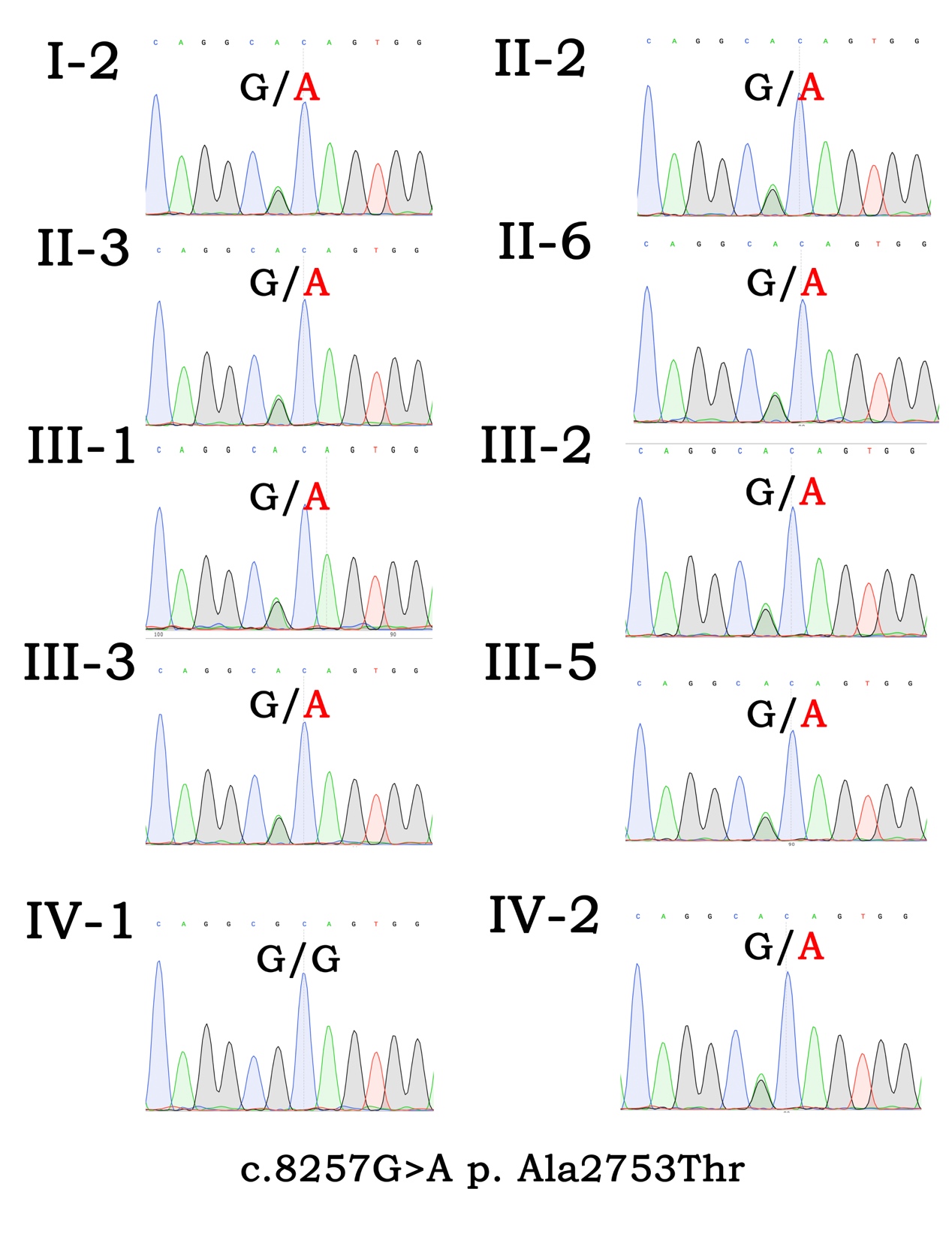


**Figure S3.** Sanger sequencing of c.8257G>A p. Ala2753Thr in Family FY-140.

Supplement: Supplementary file 3 — Additional file 3: Figure S3. Sanger sequencing of c.8257G>A p. Ala2753Thr in Family FY-140. [file 12920_2022_1269_MOESM3_ESM.docx]
